# An Echogenic Clot Method for Thrombolysis Monitoring in Thrombotic Stroke Models

**DOI:** 10.18103/mra.v11i3.3702

**Published:** 2023-03

**Authors:** Dalton E. Carter, Tao Peng, Melanie R. Moody, Shao-Ling Huang, David D. McPherson, Melvin E. Klegerman

**Affiliations:** 1 Department of Internal Medicine, Division of Cardiovascular Medicine, University of Texas Health Science Center at Houston, Houston, Texas 77030, U.S.A.

## Abstract

To demonstrate thrombolytic efficacy of a tissue plasminogen activator (tPA)-loaded echogenic liposome (TELIP) formulation in a rabbit thrombotic stroke model (the most relevant animal model for evaluation of directed thrombolytic therapy for ischemic stroke), we sought to develop a means of monitoring thrombus dissolution quantitatively by ultrasound imaging methods. We hypothesized that a gas-free ultrasound contrast agent can be incorporated into blood clots at a concentration that does not affect the tPA-mediated clot dissolution rate, while enabling quantitative assessment of the clot dissolution rate. Clots were formed from a mixture of whole rabbit blood, 1 M calcium chloride, human thrombin and varying amounts of microcrystalline cellulose. Washed clots in tubes were weighed at 30, 60 and 90 minutes after addition of recombinant tPA (rtPA) in porcine plasma (100 μg/ml). Clot echogenicity at each time point was assessed using a Philips HDI 5000 ultrasound system using an L12-5 linear array probe. Recorded Images underwent videodensitometric analysis that converted image reflectivity to mean gray scale values (MGSV). We found that 1.12 mg/ml of microcrystalline cellulose in rabbit blood clots (0.2 ml) provided optimal echogenicity without affecting clot dissolution rates (0.3-0.6 mg/min.) caused by rtPA. The clot dissolution rate measured by videodensitometric analysis of the echogenic clots agreed well with that determined by mass loss measurements (0.28% 0-time value/minute). This method will be important for demonstrating *in vivo* efficacy with potentially decreased hemorrhagic effects provided by directed tPA vehicles relative to systemic administration of the free thrombolytic.

## Introduction

Stroke ranks second as a cause of death worldwide and third as a cause of lost disability-adjusted life-years in high-income countries. In the United States, 87% of strokes are caused by focal cerebral ischemia due to arterial occlusion (ischemic stroke)^1,2^ . Ischemic stroke is the most common acute neurologic illness. Stroke-related costs in the United States came to nearly $53 billion between 2017 and 2018^2^. The introduction of recombinant tissue plasminogen activator (rtPA, Alteplase) as a clinical thrombolytic agent for acute ischemic stroke (AIS) 25 years ago was a breakthrough in the treatment of strokes caused by clots in the carotid and cerebral arteries ^3-5^. However, free tPA’s hemorrhagic side effects and the difficulty treating clots in the carotid and cerebral arteries have limited the usefulness of free tPA in treating acute ischemic stroke ^6-8^. Ultrasound has been found to improve the effectiveness of rtPA, but hemorrhagic side effects, which can be fatal, continue to limit optimization of the protocols ^9^. Because rtPA produces low rates of complete recanalization and increased rates of symptomatic intracerebral hemorrhage (ICH), only 2-5% of acute ischemic stroke patients in the United States are given intravenous rtPA treatment ^10-13^. The use of intracranial Doppler ultrasound treatment as an adjunct to intravenous rtPA was shown to increase recanalization (49% vs. 30% rtPA only, p = 0.03) within 2 hours of rtPA administration in acute ischemic stroke patients in the CLOTBUST study ^14^. However, the improved recanalization did not persist, as indicated by neurological outcome 3 months after treatment (42 vs. 29%, p = 0.20).

We have developed a novel therapeutic carrier, intrinsically echogenic liposomes (ELIP), that can serve not only their original purpose as an ultrasound contrast agent, but also as a vehicle for ultrasound-triggered controlled drug release ^,15,16^. In addition, we have successfully loaded rtPA into these carriers, creating an ultrasound-controlled thrombolytic drug-delivery nanotechnology ^17^. TELIP’s thrombolytic activity is comparable to that of free tPA and is enhanced by both continuous wave and pulsed Doppler ultrasound ^17-19^. A preliminary in vivo study using a rabbit aorta clot model demonstrated that Doppler ultrasound enhances TELIP thrombolysis significantly in 15 minutes ^20^. A follow-up study using this model showed that TELIP has the thrombolytic efficacy of locally delivered rtPA ^21^. This formulation may overcome the longtime problems of tPA treatment of acute ischemic stroke and significantly improve thrombolytic therapy by combining the self-targeting TELIP construct with ultrasound tPA activity enhancement^22^.

An appropriate approach to establishing the thrombolytic efficacy of TELIP, with and without transcranial pulsed Doppler ultrasound, relative to free rtPA in the treatment of acute ischemic stroke would be to test it in a rabbit embolic stroke model. This model was used to optimize rtPA therapy for acute ischemic stroke three decades ago ^23^. Treatment efficacy is evaluated by in situ measurement of thrombus dissolution rate and middle cerebral artery (MCA) flow rate, as well as behavioral measures of stroke recovery and brain infarct volume. The original study employed radiolabeled thrombi monitored by quantitative scintigraphic analysis. Magnetic resonance imaging (MRI) is currently the method of choice for monitoring the rate of *in vivo* thrombus dissolution in real time, but requires specialized instrumentation and experienced investigators.

To devise a method for monitoring thrombus dissolution that is relatively simple and inexpensive, while avoiding the use of radioactive materials, we aimed to demonstrate the feasibility of monitoring clot dissolution rates using quantitative ultrasound imaging techniques. This was accomplished by videodensitometric analysis of ultrasound contrast agent-impregnated clot sonograms at various times. This method has the additional advantage of utilizing the same equipment required to insonify the thrombus during TELIP administration.

## Materials and Methods

### Optimization of recombinant tPA-Mediated Clot Dissolution

Clots were formed in 1.5 ml Eppendorf tubes (Fisher Scientific, Waltham, MA) by mixing 100 μl whole rabbit blood with 25 μl 1 M calcium chloride, 50 μl 0.02 M phosphate-buffered saline, pH 7.4 (PBS), and 25 μl thrombin (2.5 U), and incubating at 37° C for 2-18 hours. Varying concentrations (60, 80 or 100 μg/ml) of recombinant tissue plasminogen activator (rtPA; Alteplase, Genentech, South San Francisco, CA) in 500 μl porcine plasma (as a source of plasminogen) was added to pre-weighed clots; replicate (3X) clots were washed with PBS and weighed with an OHAUS analytical balance (OHAUS Corp., Pine Brook, NJ) at 30, 60 and 90 minutes after addition of rtPA. The rtPA concentration giving the best linear clot dissolution rate over 90 minutes was chosen for subsequent studies.

### Determination of Non-Interfering Ultrasound Contrast Agent Dose

The echogenicity of microcrystalline cellulose (MCC; Sigma-Aldrich, St. Louis, MO) suspensions was confirmed by intravascular ultrasound (IVUS). Recorded images were subjected to videodensitometric analysis that converted image reflectivity to mean gray scale values (MGSV) as previously described ^24,25^. Varying volumes (10-30 μl) of a 7.5 mg/ml MCC suspension in water were added during clot formation, with reduction of the PBS volume by a corresponding amount. Clot echogenicity at each time point was assessed with a Philips HDI 5000 ultrasound system using an L12-5 linear array probe ^21^ positioned laterally to tubes placed in an anechoic chamber. Videodensitometric analysis was performed. Alteplase (100 μg/ml)-mediated clot mass loss rates were determined as described above.

### Determination of Clot Dissolution Rates Based on Echogenicity Loss

Clot echogenicity loss was measured as MGSV decrease and % reduction (using 0.225 mg MCC/clot), which was compared with the clot mass loss rate.

### Statistics

Linear regressions, including fit equations and variance analyses of the individual points, were performed with SigmaPlot 10.0 software (Systat Software, Inc., Richmond, CA).

## Results

**Figure 1.**
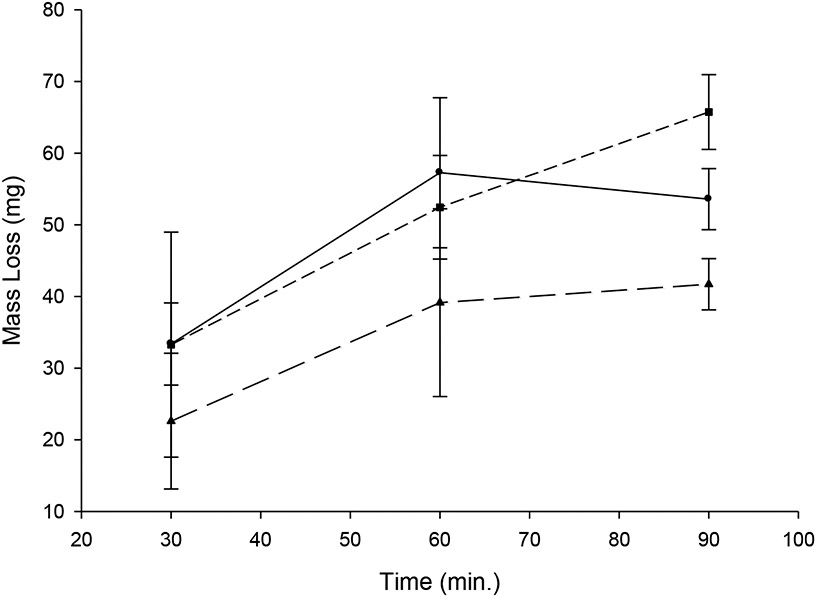
Optimization of tPA concentration. Comparison of three concentrations: circles, solid line, 60 μg/ml; triangles, long dash line, 80 μg/ml; squares, short dash line, 100 μg/ml. Each point is the mean of 3 determinations; bars = SD.

Of the rtPA concentrations added to clots in 500 ml porcine plasma, 100 μg/ml provided the best linear dissolution rate (Fig. 1), which was reproducible.

**Figure 2.**
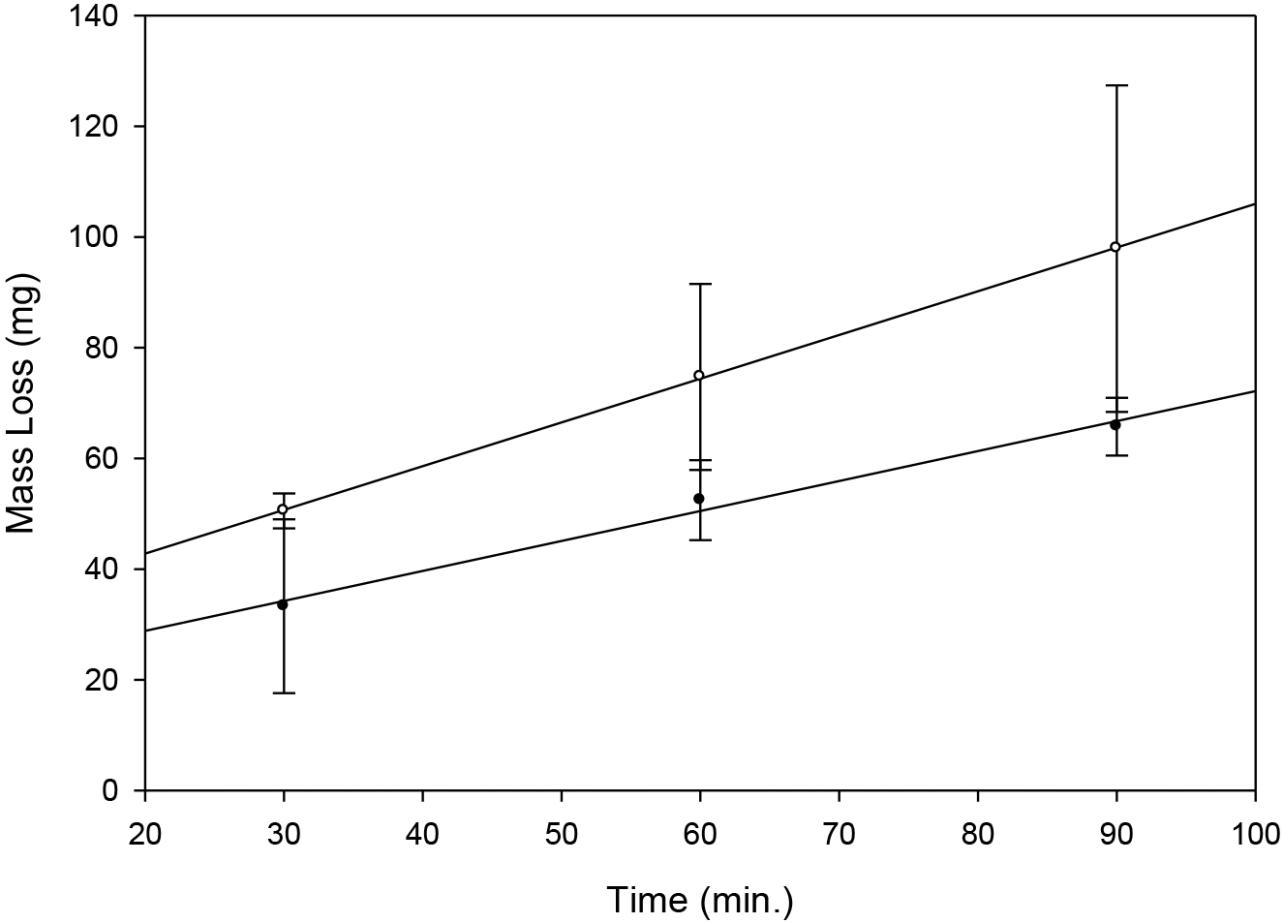
Reproducibility of clot mass loss rate with 100 μg/ml of rtPA. Filled circles: y = 0.54x + 18.0, r = 0.838; open circles: y = 0.78x + 26.1, r = 0.999. Each point is the mean of 3 determinations; bars = SD; p > 0.1 for comparison of means at each time point.

Two repetitions of the dissolution curve with 100 μg/ml rtPA exhibited good linearity and were nearly parallel (Fig. 2). Differences between clot mass loss means were not significant (p > 0.1) at each time point.

**Figure 3.**
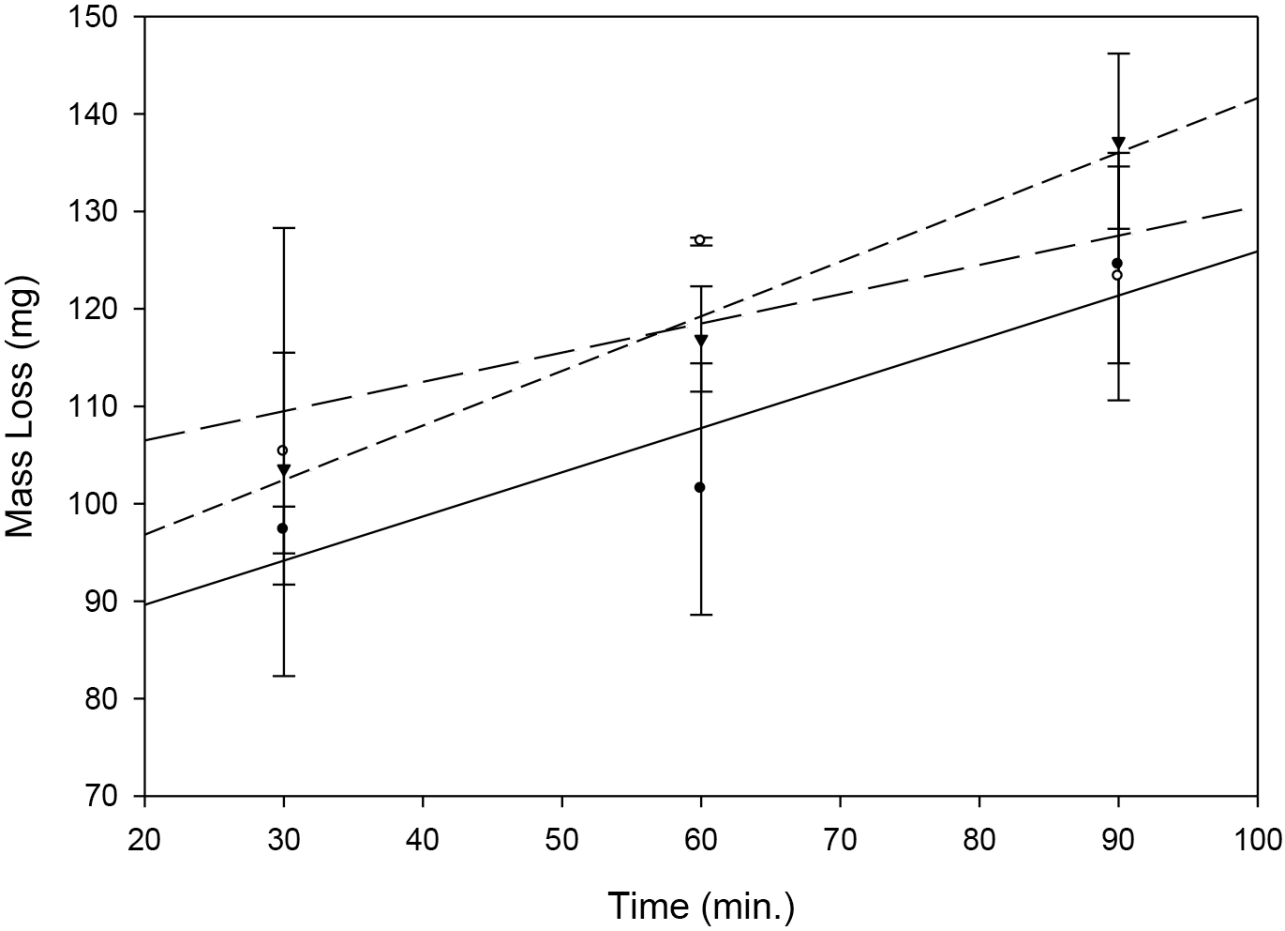
Comparison of tPA-mediated clot dissolution with addition of varying amounts of MCC stock suspension. Each point is the mean of 3 determinations; bars = SD. Solid circles, solid line: 10 μl (0.075 mg), y = 0.453x + 80.6, r = 0.929; open circles, long dash line: 20 μl (0.150 mg), y = 0.3x + 100.5, r = 0.778; inverted solid triangles, short dash line: 30 μl (0.225 mg), y = 0.56x + 85.6, r = 0.993.

**Figure 4.**
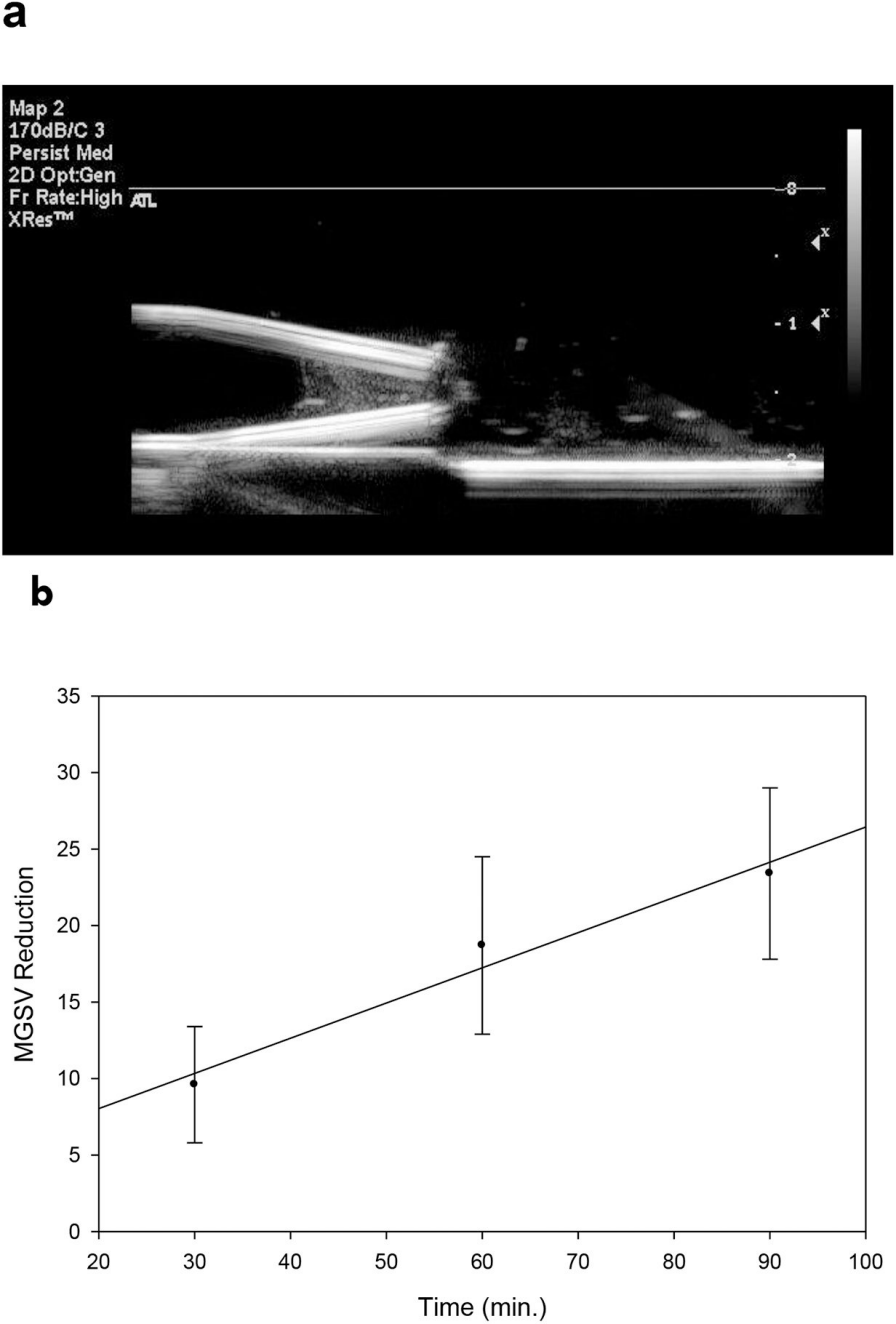
Measurement of clot dissolution by ultrasound imaging. a. Sonogram of MCC-doped clot (0.225 mg/clot). b. Monitoring of tPA-induced clot dissolution by quantitative image analysis; 0.225 mg MCC, 100 μg/ml of rtPA. Each point is the mean of 3 determinations; bars = SD.

Addition of varying amounts of the stock microcrystalline cellulose (MCC) suspension, up to 30 μl (0.225 mg) per clot, allowed clot dissolution rates similar to undoped clots (Fig. 3). Addition of 30 μl stock MCC suspension provided suitable echogenicity (Fig. 4a), which decreased linearly after addition of tPA with time, for up to 90 minutes (Fig. 4b).

**Figure 5.**
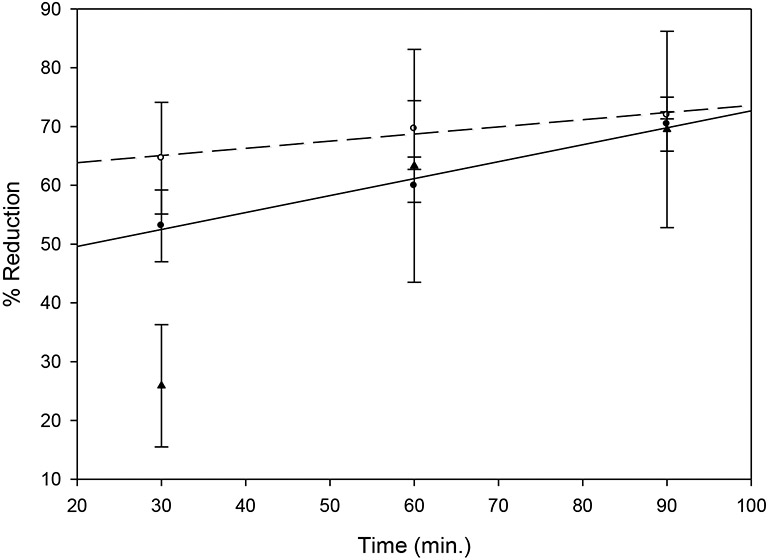
Clot dissolution determined by ultrasound image analysis, relative to dissolution rates measured by clot mass loss (CML); 0.225 mg MCC, 100 μg/ml of rtPA. Bars = Mean ± SD, n = 3. Closed circles, solid line: CML1, y = 0.29 x + 43.8, r = 0.992; open circles, dashed line: CML2, y = 0.12 x + 61.4, r = 0.978; triangles, no line: ultrasound imaging.

When clot mass loss and MGSV data were converted to percent clot reduction as a means of comparison, serial videodensitometric measurements of tPA-treated clots yielded dissolution rates at 60 and 90 minutes that were very similar to those found by clot mass loss determinations (Fig. 5).

The dissolution rate measurements of clot mass loss by each method were similar. Divergence was noted below 60 minutes for both measurements. This may indicate increased variability at earlier stages of the clot dissolution process.

## Discussion

In this study, we have shown that a) a soluble ultrasound contrast agent (microcrystalline cellulose) can be incorporated into blood clots without affecting the rate of tPA-induced mass loss; b) the MCC-doped clots can be visualized by conventional ultrasound imaging methods; c) tPA-induced clot dissolution can be monitored quantitatively by videodensitometric techniques; and d) clot dissolution rates determined by US monitoring are similar to those determined by clot mass loss measurements. Thus, we have created a method for monitoring tPA-induced thrombus dissolution suitable for real-time measurements in a rabbit thrombotic stroke model.

Clinical studies have demonstrated that use of focused ultrasound plus a thrombolytic agent improves thrombolysis for stroke and acute myocardial infarction ^14,26,27^. This may be due to improved diffusion that promotes transport of drugs into the thrombus, reformation and opening of the fibrin matrix that enhances drug diffusion, clearing of fibrin polymers that increases the surface for drug interaction, or direct effects on binding of the agent to fibrin ^28^. Ultrasound produces cavitation, which allows large molecules and particles to penetrate cells (sonoporation) ^29^, and this property is actively being investigated for drug and gene delivery ^30-34^. Addition of a contrast agent into a carrier as an ultrasound nucleation agent can lower the threshold for these ultrasound bioeffects ^35,36^.

We have demonstrated that clinical Doppler US increases TELIP thrombolysis, both *in vitro* and *in vivo* ^18,19^. In a preliminary study using a rabbit aorta thrombolysis model, we showed complete recanalization 15 minutes after TELIP administration in rabbits receiving 5.7 MHz pulsed Doppler ultrasound (MI = 0.43) vs. 60% mean recanalization in rabbits receiving TELIP only ^20^. A follow-up study, comparing TELIP to established clinical thrombolytic protocols, established that TELIP was at least as effective as free rtPA, with and without Definity cavitation, in terms of maximum percent recanalization within a 30-minute period and rate of total recanalization ^24^. Pulsed ultrasound was used in that study to extend previously observed thrombolytic potentiation from continuous wave ultrasound to clinically relevant conditions.

The Zivin rabbit stroke model ^23^ provided the basis for optimization of rtPA protocols for clinical ischemic stroke therapy ^37^. We have demonstrated the efficacy of TELIP in a rabbit abdominal aorta thrombus model, but a demonstration of both efficacy and safety in a stroke model is a necessary next step in preclinical development of the formulation. The larger vessel size in rabbits should provide a better projection of clinical TELIP efficacy than commonly used rat embolic stroke models, although rat ischemic stroke models have been sufficient to demonstrate free tPA efficacy^38,39^.

In addition, this rabbit thrombotic stroke model was used to establish the kind of safety and efficacy information that is required for translational product development and was shown to be clinically relevant ^37^. Such a study would provide information needed to extend these findings to identify safety and efficacy of our novel therapeutic carrier, to define our ability to utilize ultrasound as a therapeutic adjunct for improved thrombolysis without increasing cerebral hemorrhage, and to perform baseline trials allowing the TELIP formulation and protocol to be extended into the clinical arena.

To that end, we have demonstrated in this study that ultrasound imaging can be employed quantitatively to monitor clot mass loss in real time during plasminogen activator-induced thrombolysis. This method must now be validated *in vivo*, preferably in Zivin’s rabbit thrombotic stroke model. Such validation will add a convenient, relatively inexpensive option to the currently employed radiotracer and MRI methodologies.

## Conclusion

We have devised a method of monitoring thrombolytic-induced clot mass loss by ultrasound imaging using a non-gas containing contrast agent introduced into the thrombus and have shown that dissolution rates in the presence of tPA measured by this method agreed with rates determined by clot mass loss measurements. This method will be useful in determining the efficacy of nanoparticulate tPA formulations in rabbit ischemic stroke models where MRI measurement is not available.
